# Bench to Bedside: Modelling Inflammatory Arthritis

**DOI:** 10.1093/discim/kyac010

**Published:** 2022-11-23

**Authors:** Chiamaka I Chidomere, Mussarat Wahid, Samuel Kemble, Caroline Chadwick, Richard Thomas, Rowan S Hardy, Helen M McGettrick, Amy J Naylor

**Affiliations:** Rheumatology Research Group, Institute of Inflammation and Ageing, University of Birmingham, Birmingham, B15 2TT, UK; Rheumatology Research Group, Institute of Inflammation and Ageing, University of Birmingham, Birmingham, B15 2TT, UK; Rheumatology Research Group, Institute of Inflammation and Ageing, University of Birmingham, Birmingham, B15 2TT, UK; Biomedical Services Unit, University of Birmingham, Birmingham, B15 2TT, UK; Biomedical Services Unit, University of Birmingham, Birmingham, B15 2TT, UK; Institute of Clinical Sciences, University of Birmingham, Birmingham, B15 2TT, UK; Rheumatology Research Group, Institute of Inflammation and Ageing, University of Birmingham, Birmingham, B15 2TT, UK; Rheumatology Research Group, Institute of Inflammation and Ageing, University of Birmingham, Birmingham, B15 2TT, UK

**Keywords:** Rheumatoid arthritis, TNF, murine models of arthritis, 3Rs, clinical score

## Abstract

Inflammatory arthritides such as rheumatoid arthritis are a major cause of disability. Pre-clinical murine models of inflammatory arthritis continue to be invaluable tools with which to identify and validate therapeutic targets and compounds. The models used are well-characterised and, whilst none truly recapitulates the human disease, they are crucial to researchers seeking to identify novel therapeutic targets and to test efficacy during preclinical trials of novel drug candidates.

The arthritis parameters recorded during clinical trials and routine clinical patient care have been carefully standardised, allowing comparison between centres, trials, and treatments. Similar standardisation of scoring across *in vivo* models has not occurred, which makes interpretation of published results, and comparison between arthritis models, challenging. Here, we include a detailed and readily implementable arthritis scoring system, that increases the breadth of arthritis characteristics captured during experimental arthritis and supports responsive and adaptive monitoring of disease progression in murine models of inflammatory arthritis.

In addition, we reference the wider ethical and experimental factors researchers should consider during the experimental design phase, with emphasis on the continued importance of replacement, reduction, and refinement of animal usage in arthritis research.

## Graphical Abstract

**Graphical Abstract:**
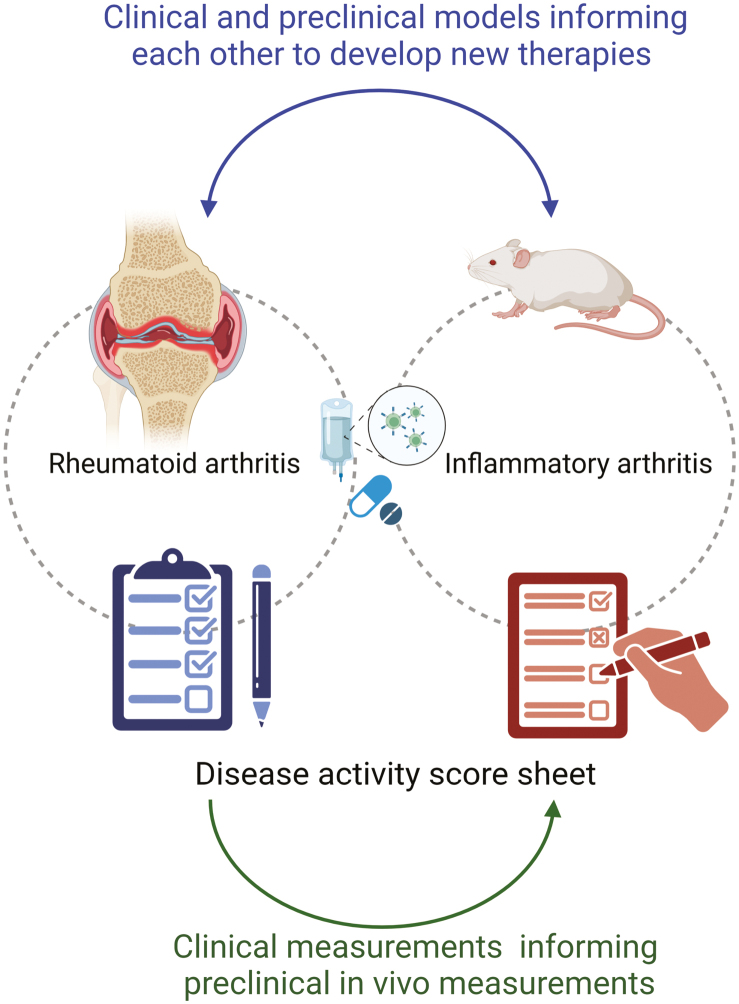

## Introduction

Inflammatory arthritides are a major cause of disability. One form of inflammatory arthritis alone (rheumatoid arthritis) affects ~1% of the UK population [1]. Biological treatments that target leukocytes or their cytokine products have improved patient outcomes (meta-analysis and systematic review [2]:), but they do not reverse tissue damage nor do they cure disease. Pre-clinical animal models of inflammatory arthritis (IA) have proven an invaluable tool in dissecting the cellular and molecular mechanisms underpinning disease, and are widely used to validate the efficacy of new therapeutic targets and compounds.

Analysis of synovial tissue from patients with rheumatoid arthritis (RA) has revealed a multitude of cell types, both leukocyte subsets and different types of tissue resident stromal cells [3–6], that are involved in the onset, progression and pathogenesis of the disease and are themselves therapeutic targets. Moreover, such studies have highlighted the level of disease heterogeneity within patient cohorts – describing different disease pathotypes based on the cellular composition of the joint and response to therapy [7–9]. Most recently the importance of specific subpopulations of tissue-resident cells, including macrophages, endothelial cells and fibroblasts, have been identified that drive inflammation, damage, and remission [5, 10–13]. This work has enabled the identification of pathogenic cell subtypes, and in some cases pathogenic signalling pathways, which present novel drug targets.

The nature of the interactions identified to date highlights the complex cellular crosstalk involved in driving inflammatory processes. Such cellular complexity is extremely difficult to achieve using conventional *in vitro* models, which are often limited to two or three cell types cultured under normoxic conditions in the absence of fluid dynamics (e.g., conditions that mimic blood flow or interstitial fluid flow). Whilst the development of organoid culture systems, hypoxic chambers and microfluidic multi-cell, multi-layered systems are becoming more widely available for routine use, we are still a long way from the fully human “joint-on-a-chip” model that could replace the use of animal models of disease. Furthermore, almost all the world’s medicines regulatory organisations require pre-clinical, animal-based evidence of therapeutic efficacy, as well as pharmacokinetic and pharmacodynamic profiles, and toxicology information prior to new compounds being “tested” in humans during clinical trials. Thus, it remains crucial that we have robust, reproducible, and refined animal models of inflammatory arthritis that model all aspects of human disease pathology.

Murine models of inflammatory arthritis have played a significant role in identifying novel biological agents for clinical use in treating patients with RA. Indeed, several in-depth reviews chart the development of the family of TNF-inhibitors (etanercept, adalimumab, infliximab), followed by anti-IL-6R targeting with tociliziumab, anti-leukocyte strategies (e.g., anti-CD20 - Rituximab, anti-CTLA-4 - abatacept) and more recently JAK inhibitors [14–17]. In most cases, researchers have favoured using CIA to validate the therapeutic efficacy of such agents. For example, TNFα inhibitors have been reported to delay the onset of disease, reduce clinical score, or reduce paw thickness in studies by different groups [18–20]. Whilst all groups use a numerical scoring system to assess inflammation/disease severity this varies between studies, with some focusing solely on the joints, assigning a score 0-2, 0-3, or 0-4, and others focusing on a combined score across the paw, tail, nose and ear [18–21]. In this article we include a detailed and readily implementable arthritis scoring system, that, if widely adopted, could form the basis of a more standardised system of data collection.

## Animal models of inflammatory arthritis

Animal models of inflammatory arthritis can be broadly divided into monoarthritic models affecting one joint, or polyarthritic models affecting two or more joints. These models occur by one of two broad mechanisms:

(i) Spontaneous onset of chronic disease driven by genetic manipulations e.g., TNF^ΔARE^, hTNF Tg, K/BxN [22–24].
(ii) Inducible resolving disease triggered either by injection of antigens e.g., antigen-induced arthritis – AIA and collagen-induced arthritis – CIA [25, 26]; or autoreactive antibodies e.g., serum transfer induced arthritis – STIA – transfer of serum containing autoantibodies from K/BxN mice [24].

Whilst the models currently in use are well characterised (**Table 1**) and share some histological or immunological characteristics with the human disease, none truly represent the heterogeneity and chronicity of RA. For example, AIA driven by methylated BSA or STIA are acute resolving models of arthritis lasting 5 or ~20 days respectively, which are predominantly driven by monocyte or neutrophil infiltrates. They have the advantage of being inducible on almost any strain of mouse with >98% penetrance of disease [27], making them highly consistent and reliable models that require small groups of animals (typically 4-6 mice per group depending on the expected effect size). By contrast, in the K/BxN transgenic mouse strain disease is driven by an autoimmune response leading to the production of glucose-6-phosphate isomerase (G6PI) autoantibodies and onset of detectable clinical symptoms at ~4-5 weeks of age [24, 28].

**Table 1:** Widely used models of inflammatory arthritis, and their characteristics: Models of RA vary considerably in their pathogenesis and disease course, depending on the species and genetic background of the rodent strain used. Spontaneous arthritis models occur in susceptible rodents due to genetic modifications or spontaneous mutations. Inducible forms of inflammatory arthritis are triggered either through break of tolerance or via transfer of autoantibodies or inflammation-inducing noxious stimuli. The aetiology of disease influences the disease characteristics and the cellular composition of the resulting inflammation.

Model	Species	Susceptible genetic background(s)	Disease Characteristics	Cellular composition of inflammatory infiltrate (major players).	References
**“Spontaneous” Arthritis Models**					
**HLA-B27 transgenic animals**	Mice, rats	B27 heavy chain transgene	Arthritis, colitis, ankylosing spondylitis	T cells	McMichael and Bowness, 2002 [29].
**K/BxN arthritis**	Mice	TCR transgenic on NOD	Arthritis due to transgenic encoded glucose 6	Neutrophils, macrophages, Mast cells, T cells, B cells	Kouskoff et al. 1996 [24]; Punzi et al. 2016 [30]; Wipke and Allen. 2001 [31]; Solomon et al. 2005 [32]; Lee et al. 2002 [33].
**ZAP-70-mutant SKG mouse**	Mice	Balb/c, Spontaneous mutation in ZAP70	Erosive arthritis with autoreactivity	T cells	Sakaguchi et al. 2003 [34].
**IL-1 receptor antagonist knockout mice**	Mice	Balb/c, ILRa deficiency	Arthritis	CD4^+^ T cells	Iwkura. 2002 [35]
**Gp130 IL-6R mutated mouse**	Mice	C57BL/6, IL-6R mutation	Arthritis	CD4^+^ T cells	Jones et al. 2013 [36]; Silver and Hunter. 2010 [37]
**TNF** ^**dARE**^	Mice	TNFdARE on C57BL/6.	Arthritis, inflammatory bowel disease, psoriasis	Synovial fibroblasts	Kontoyiannis et al. 1999 [22]
**TNF Tg**	Mice	TNF Tg on C57BL/6	Arthritis, inflammatory bowel disease, psoriasis	Synovial fibroblasts	Keffer et al. 1991 [23]
**“Induced” Arthritis Models**					
**Pristane Induced arthritis**	Mice, rats	MHC, non-MHC loci on chromosome 1,4,6,12,14 BALB/c, DBA and C3H background	Generalised inflammation. Chronic arthritis and erosive arthritis in peripheral joints	T cells	Benson et al. 2018 [38].
**Collagen Type II (heterologous or homologous CII in CFA)**	Mice	MHC (q and r), non-MHC loci	Erosive polyarthritis In peripheral joints	T cells, B cells	Courtney et al. 1980 [26] Nakajima et al. 1993 [39].
**Collagen-antibody induced arthritis**	Mice, rats	Balb/c, DBA/1, C57Bl6	Self-limiting arthritis	T cells, B cells, macrophages	McNamee et al. 2015 [40]; Nndakumar et al. 2003 [41].
**Fibroblast transferred SCID mouse**	Mice	Immunodeficient SCID mouse	Sustained destructive arthritis	Synovial fibroblasts	Noss and Brenner. 2008 [42]; Frey et al. 2018 [43].
**Ovalbumin (OVA) TCR transfer**	Mice	BALB/c, C57Bl6, OVA peptide 323–339 complexed with the MHC class II molecule I-A	Polyarthritis	T cells, B cells, Macrophages, Neutrophils, CD25^+^Foxp3^+^Tregs	Attridge and Walker. 2014 [44]; Maffia et al. 2004 [45]; Brackertz et al. 1977 [46].
**Antigen induced (methylated bovine serum albumin (mBSA))**	Mice	C57BL, BALB/c	Monoarthritis	T cells and CD4^+^CD25^+^T cells	Li and Schwarz. 2003 [25] Frey et al. 2005 [47].
**Streptococcal cell wall (SCW) induced**	Mice, rats	BALB/c, Lewis’s rats, non-MHC genes	Erosive Polyarthritis	T cells, B cells, Macrophages	Bevaart et al. 2010 [28].
**K/BxN serum transfer arthritis**	Mice	C57Bl/6, BALB/c	Resolving, non-erosive arthritis. Multiple repeat injections of serum can induce chronic, erosive disease	Neutrophils	Kouskoff et al. 1996 [24] Christensen et al. 2016 [27].

Of all the available options, the collagen-induced arthritis (CIA) model most closely resembles the pathological changes seen in human RA and has been used successfully in the discovery and development of disease-modifying anti-rheumatic drugs (DMARD), reviewed by Luan et al [48]. Given this clinical and historical backdrop, collagen-induced arthritis is considered the field’s gold standard and is often required for pre-clinical studies and by funders. In practice, this model is challenging to use because it is highly variable in day of onset and severity, heterogeneous in the number and pattern of joints affected, and with limited disease penetrance that is strain dependent (40-60% on the C57BL/6 or 60-80% on the DBA-1 background [49]). As such, it requires more mice per experimental group to achieve statistical power, with minimum group sizes of approximately 10-15 to allow for variance and experimental attrition. In particular, the C57BL/6 strain, widely used in research for the array of genetically engineered populations shows a particularly low susceptibility [50]. Further limitations arise due to its lack of chronicity, with disease beginning to resolve from day 10-14 post onset and with evidence of fibrosis and repair, which are not seen in the human disease [51]. Of note, the C57BL/6N.Q mouse strain (in which the MHC class II arthritis susceptibility locus Aq is expressed) is more susceptible to CIA disease induction and demonstrates more robust chronicity as compared to the frequently used C57Bl/6J background [52]. As such, it may be more suitable for studies involving genetically modified strains.

Balancing the scientific requirements of the model (including aetiology, similarity to the human disease, specific pathway/cell-type involvement) with the practical experimental elements (e.g., number of animals required for statistical significance, genetic background of the experimental animals available) and the welfare and ethical costs (degree of distress and lasting harm caused) is extremely challenging. In addition, the differences between the routinely used arthritis models represent a fundamental challenge to researchers attempting to translate observations in rodents to clinical therapies for patients.

**Figure 1:**
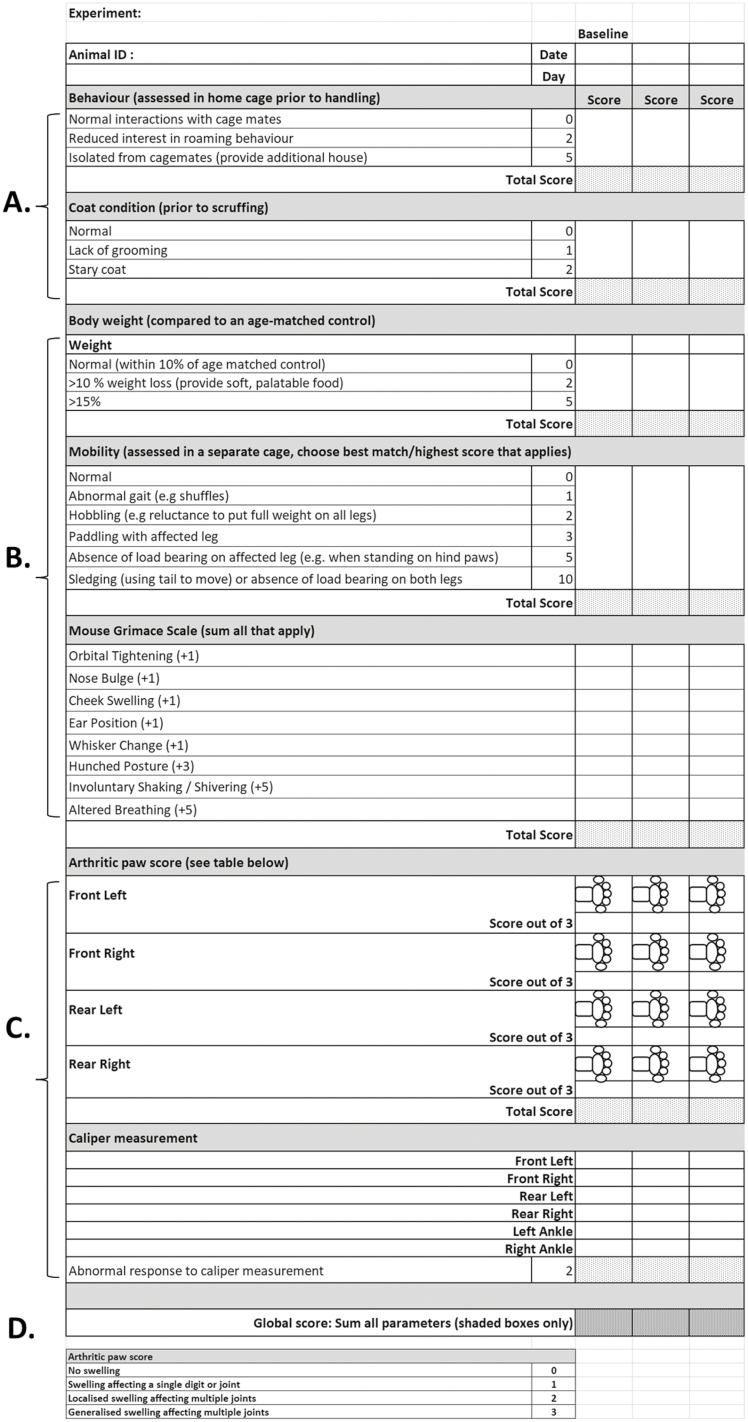
Example score sheet for inflammatory arthritis monitoring. **(A)** First, behaviour and coat condition are observed in home cage and prior to any handling. **(B)** Subsequently, weighing of individual animal followed by transfer to a new, clean cage (clear of any housing or bedding) to allow observation of mobility. Evidence of “grimace” as described in the Mouse Grimace Scale [53] can be recorded at this stage, if not already. **(C)** Finally, each paw of the restrained mouse is observed, and swollen regions shaded on the paw schematic. A score is then given of between 0 – 3, based on the number of joints/regions shaded (0, represents no visible swelling, 1 = 1 or 2 affected joints; 2 = multiple affected joints; 3 = generalised swelling across the paw). Whilst restrained, calliper measurements can be taken of the front and rear footpads and of the rear ankles (hock joint). **(D)** The “arthritic paw score” or “clinical score” can then be calculated. The sum of scores from each of A, B, and C give the “global score”, thus comprising both clinically evident arthritis and the clinically evident extra-articular manifestations of disease.

In this perspective, clinical practice is compared to IA modelling *in vivo*, to highlight the need for more robust, reproducible data collection and reporting procedures to ensure consistent high-quality data are obtained and the translational value of such studies. We describe a detailed and readily implementable arthritis scoring and welfare assessment protocol (**Figure 1**) that supports responsive and adaptive monitoring of disease progression in murine models of inflammatory arthritis, as well as informing analgesia treatment decisions and enabling early identification of appropriate humane endpoints.

For the purposes of this discussion, and where appropriate, we focus discussions on two widely used IA models: STIA and CIA. Throughout, we reference the wider ethical and experimental factors researchers should consider as they design and conduct such studies to support more translation of research findings into clinical practice. Finally, we discuss the continued importance of replacement, reduction, and refinement of animal usage in arthritis research and the options currently available to researchers in this field.

## Generating robust and reproducible data from animal models of inflammatory arthritis

Central to ensuring the relevance and translatability of *in vivo* arthritis studies are the choice of model and the experimental design. Model choice has been reviewed extensively by Vincent et al [54]. and an overview summary of the available models and their characteristics is provided in **Figure 1**. Pragmatic and scientific decisions must be balanced with ethical considerations to ensure that the correct model is chosen to answer the most relevant scientific question and that the data generated are robust, conclusive, reproducible, and translational.

When considering translation, thought should be given to whether prophylactic or therapeutic treatment is most relevant. All models can, theoretically, be used to measure efficacy of either treatment regime, however those with variable onset and penetrance are less suited to prophylactic interventions, due to the difficulty in establishing the effect size in small cohorts. Additionally, consideration should be given to the timing of prophylactic, subclinical and therapeutic interventions in terms of the phase of disease in the animal and how this compares to the phases of human inflammatory arthritis.

It should be noted that monoarthritis models, such as antigen-induced arthritis are generally considered to be less severe than the classical polyarthritic models (e.g., K/BxN or collagen-induced models). As such, careful consideration needs to be given to the use of polyarthritic models based on the specific disease mechanism and experimental question, with these models only used as translational tools if there is strong evidence supporting this requirement [54, 55]. The scoring system detailed here (Figure 1) is suitable for comparison and monitoring of polyarthritic models only.

### Cohort choice

As with the arthritic diseases that IA models such as STIA and CIA aim to replicate, genetic and environmental factors can influence disease onset and severity. For instance, the microbiome of the gut, lungs and oral cavity have all been linked to various aspects of RA pathogenesis, including onset of disease [56–61]. The same holds true for inducible IA models, and substantial variation has been observed in the incidence of arthritis between research centres. This is particularly relevant for models such as CIA where onset is dependent on the breaking of tolerance to self-antigen. Given this, we recommend that purchased animals are acclimatised for at least 10 days to any new animal facility prior to arthritis induction protocols. Despite allowing for an acclimatisation period, in our experience DBA-1 strains from different providers continue to vary widely in their incidence of CIA and it is important that each centre undertaking arthritis studies understands and optimises this at the beginning of any programme of work. In addition, the degree of arthritis induced via K/BxN serum transfer varies markedly depending on the batch of serum used and the colony from which it was obtained. For this reason, batch-testing of serum is required prior to embarking on any experiment to identify the correct dose and booster-dose requirements.

One factor of importance in the design of IA studies in mice is the selection of biological sex. Whilst both sexes of mice are susceptible to many IA models, early reports that CIA induced with homologous collagen II developed exclusively in male mice [62] has encouraged most research groups to conduct pre-clinical arthritis studies using only male animals. However, RA exhibits a sex-bias, affecting 2-3 times as many women than men. Recent increased awareness of the importance of sexual dimorphism across numerous physiological processes has led the United States National Institutes of Health (NIH) in 2016 and the UK governmental funding agency (UKRI) in 2022 to instruct researchers to mitigate against sex-bias from study design, to ensure pre-clinical models are fit for purpose and translate to human disease [63–65]. This raises important cost and time implications for researchers. When determining experimental group sizes that include both sexes, care should be taken to determine the presence of sexual dimorphism in the study.

### Capturing relevant and reproducible data on clinical parameters

The American College of Rheumatology (ACR) and European League Against Rheumatism (EULAR) joint 2010 guidelines for RA involve assessing the tenderness and swelling in digits and large joints, counting the types and numbers of joints affected, recording symptom duration, and patient-reported outcomes [66–68]. These key clinical parameters underpin the level of disease burden and clinical severity experienced by patients and are used to monitor disease symptoms and response to therapy. In contrast, there is no universal data collection method to assess IA models, resulting in variation and inconsistency in the data reported by research groups. Most “score sheets” focus on the inflammatory signs of arthritis, capturing the number and patterns of swollen/red joints and may also include detailed tissue pathology [69]. In some instances, additional effort has been put in to capturing the degree of swelling and signs of pain and loss of joint function e.g described in Hawkins et al [55], but these parameters are rarely reported as experimental outcome measures in subsequent publications. To encourage such capturing and reporting of these data, we have developed detailed, model specific, assessment rubrics encompassing behavioural, welfare and disease parameters (example and workflow in **Figure 1**; model-specific versions in **Supplementary Figures 1-2**). These score sheets increase the data captured from each experiment and encourage the researcher to more accurately identify and access individual components of disease that underpin the IA mode. They also allow the research group and *in vivo* support team to develop a more thorough understanding of the normal progression of each IA model. The scoring system requires no specialist equipment and captures behavioural, physical, and clinical parameters that together allow a full picture of disease activity and progression to be assessed. The scoring system also provides a natural structure, process and template that can be used to train, inform, and instil confidence in staff and researchers using the IA models.

Given the emphasis that many patients with RA place on symptoms of fatigue, anxiety, stress, depression, and isolation linked to the disease (captured by the PROMS/VAS questionnaires during clinical assessment), it is important for researchers to consider these parameters as part of their assessment of mice subjected to IA. Parameters that aim to detect behavioural changes should be assessed in the home cage prior to handling to ensure that animals are in their natural surroundings and by animal handlers with extensive experience and understanding of normal mouse behaviour (**Figure 1A**). Behavioural changes, such as mice isolated from their cage mates, reduced interactions with cage mates and reduced roaming behaviour, signs of reduced grooming (scruffiness), starry coat (piloerection) and evidence of pain (Mouse Grimace Score [53]), are all indicators of discomfort, pain or distress that are encapsulated in this scoring system and that need to be carefully managed with the support of the *in vivo* research facility team.

Clinical management of RA has improved significantly, often because of data obtained from *in vivo* IA models. However even with the inflammatory symptoms reasonably well controlled, patients still report varying degrees of pain affecting their daily lives and causing immobility. Opioid-based analgesia such as buprenorphine, is frequently administered prophylactically in *in vivo* IA studies, to minimise acute and chronic pain without affecting the inflammatory responses being investigated [55]. This practice has been questioned due to concerns of the underlying action of opiate analgesics on disease pathophysiology and disease suppression [70]. Despite this, opiate analgesics remain widely used in models of IA and therefore careful consideration regarding delivery across groups is required to minimise bias. Other pharmaceutical agents such as Gabapentin, Ketorolac, Etanercept and paracetamol have also been reported to provide effective analgesia during some stages of the model, although non-opioid analgesics can have anti-inflammatory effects which can interfere with model progression and experimental outcome. This is more thoroughly discussed in Hawkins et al [55].

Whilst pain itself can be difficult to assess in mice, the level of discomfort (incapacitance) and weight distribution across paws has been determined using static weight bearing touch/incapacitance systems in rodent models of, for example osteoarthritis [71–73]. More advanced instruments have also been developed, such as dynamic weight bearing tests, that enable even faster paw identification, as well as video tracking of animals. Even in the absence of such equipment, altered or abnormal gait pattern indicative of a protective mechanism to protect an injured limb from loading or from movement-evoked pain can be observed and qualitatively assessed (**Figure 1B**). To ensure reproducibility it is important to undertake direct comparison with unaffected mice wherever possible, and that the same researcher or team of researchers carry out scoring for the duration of the study to reduce variability. To further reduce subjectivity and variability, automated, video-based systems have been developed, such as the CatWalk system, which has been successfully used to assess static and dynamic gait changes in the complete Freund’s’ Adjuvant-induced monoarthritic model [74]. Furthermore, the DigiGait Imaging System has been used in CIA studies, capturing data from multiple animals at once, and importantly showed that increased clinical scores corresponded to changes in multiple gait parameters that reflected both morphological and functional deficits [75]. Comparisons between platforms have been carried out, with variable conclusions [76, 77], highlighting the importance of careful standardisation in quantifying gait disturbances.

It should be noted that the effective use of analgesia requires animals to be re-assessed as the previous dose wears off. Outputs such as weight bearing, gait analysis, and ‘mouse grimace scale’ [53] by definition, require the animals to be experiencing pain, therefore it can be argued that these are less refined than using parameters that do not require the animal to experience pain (joint swelling, redness, number of joints/limbs affected) and that do not require a “break” in the analgesic regime to be measured. We suggest that careful and detailed monitoring of all parameters enables a detailed picture of each arthritis type to be built-up within an institution, such that the researchers and animal care staff can accurately predict disease course and provide prophylactic pain management more effectively.

To assess clinically evident swelling, each region of the fore and hind paws is assigned visually as either swollen or not swollen. Swollen regions are shaded on the scoresheet paw schematic and the number of swollen regions is converted to a score that indicates the extent of affected joints: 0, represents no visible swelling, whilst the maximum score of 3 demonstrates generalised swelling across multiple joints (**Figure 1C**). Scores across the 4 limbs are then totalled to give a score out of 12. These data are used to chart the clinical progression of disease.

Whilst uncommon in patients, measuring the degree of swelling in the affected joints using callipers is standard practice in IA models, offering further insight into disease severity. Crucially, any abnormal response to the calliper (recoiling or vocalisation) is a clear sign of ineffective pain management, which should be addressed urgently. In patients, sub-clinical joint inflammation is identified using ultrasound [78], as described below, there are various imaging methodologies that can be utilised in rodents to detect subclinical inflammation. It is worth mentioning that not all IA models exhibit swelling measurable by callipers. A particular example is the TNF^ΔARE^ mouse [22], which is characterised by progressive joint deformity and inflammatory infiltrates detectable by histology, but not by pronounced edema.

Finally, a “global score” (**Figure 1D**) is calculated from the sum of all measured parameters, including behavioural and global health parameters such as interactions with cage mates and weight. This score gives an overview of the effect of disease on the whole animal, rather than focussing purely on the joints.

**Figure 2.**
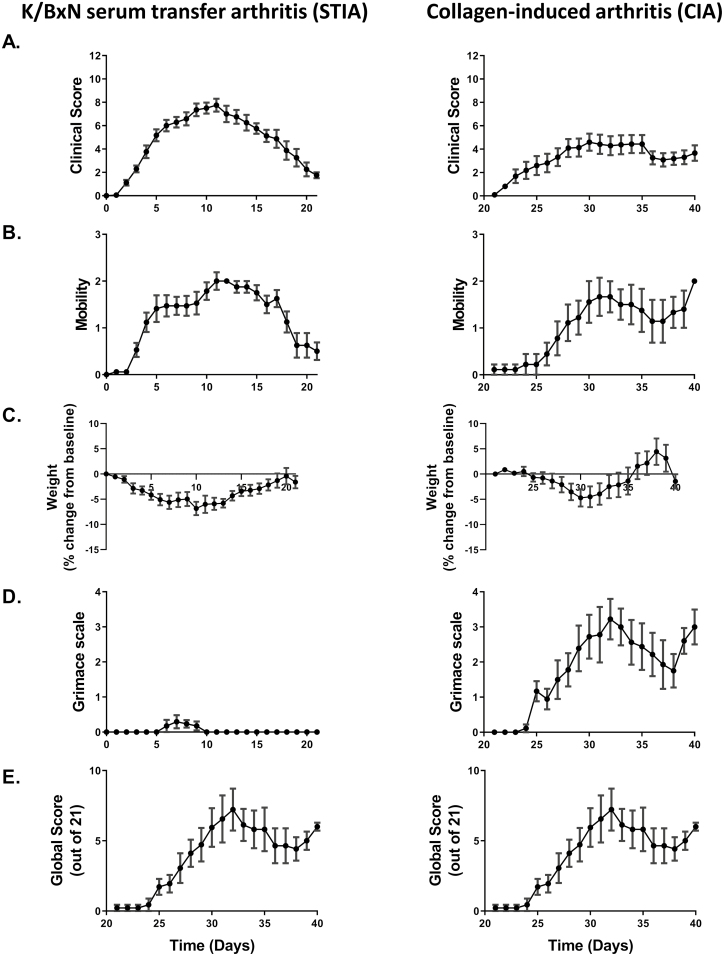
Comparison of scoring parameter outputs between two inducible models of inflammatory polyarthritis. **Left:** K/BxN serum transfer arthritis, induced in 8–10-week-old male C57BL/6J via two 100μl intraperitoneal injections of K/BxN serum. N = 17. **Right:** Collagen-induced arthritis, induced in 8–10-week-old male DBA1 mice via the protocol described in Brand *et al.* 2007 [49]. N = 17. In each case, scoring was performed using the parameters detailed above (figure 1). **(A).** Clinical score: A measure of the number of swollen joints. **(B).** Mobility: A measure of effect of arthritis on mobility. **(C).** Weight change from baseline, shown as percentage change. **(D).** Grimace scale: Evidence of grimace identified using the ‘Mouse Grimace Scale’ developed by Langford et al. 2010 [53] and scored as demonstrated on the score sheet in figure 1. **(E).** Global score: A composite measure combining the scores from all aspects of the scoring system described in figure 1. All animal experiments were performed in accordance with U.K. laws (Animal [Scientific Procedures] Act 1986) and with the approval of the local ethics committees at the University of Birmingham.

To demonstrate the type and utility of the data generated from this scoring matrix, we show example results from two commonly used models: K/BxN serum transfer arthritis (also commonly termed serum-transfer-induced arthritis or STIA) and collagen-induced arthritis (CIA) (**Figure 2**). Assessment of clinical score alone (**Figure 2A**) demonstrates the temporal differences in disease onset and progression between the two models. Note that the x-axis scale is the same for each arthritis model and is shown in increments of 5 days, but the start point varies. This is due to differences in the method of arthritis induction in the two models. In both cases, the graphs begin 2 days prior to disease onset. STIA displays rapid onset of arthritis, affecting multiple joints. Disease peaks around day 10 and rapidly resolves. Conversely, onset of symptoms in CIA is more gradual, with fewer joints affected, and plateaus over the timeframes analysed in most studies. The duration of arthritis is an important factor determining overall severity and should be minimised, commensurate with the experimental aims. Indeed, a detailed understanding of the disease time-course of each model provides an opportunity to limit the length of studies by refining the window of data collection to include only that required to understand the research question.

In these models (STIA and CIA), mobility (**Figure 2B**) largely tracks with clinically observable joint swelling. What is not interpretable from these data are whether the changes in gait and mobility are caused by a protective response to pain or by the physical impediment of joint swelling. Weight loss (**Figure 2C**) and grimace/pain face (**Figure 2D**) give an indication of the overall health of the animal and the degree of discomfort associated with their disease. In these cases, the two models deviate markedly from each other. Despite demonstrating very little evidence of grimace/pain face, mice with STIA show weight loss of approximately 5% in the early stages of disease onset. This weight loss then stabilises and returns to normal as arthritis resolves. Conversely, mice with CIA show similar weight loss during the early period of disease onset but show evidence of grimace throughout the disease course.

Combined measured parameters (including coat condition and interactions with cage-mates, not shown here) are summed to give a “global score” (**Figure 2E**). In this example, the global score is higher in the CIA despite these mice showing fewer swollen joints. Data such as these can be used to inform researchers at all stages of the design and research process. They can also aid decision-making around the most appropriate humane endpoints, timing, and duration of analgesia.

### Tools to further refine *in vivo* IA studies

**Figure 3.**
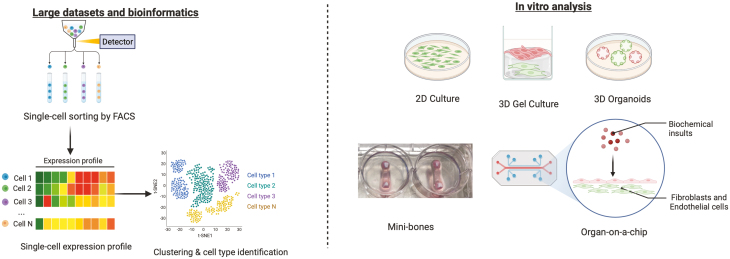
Current replacement options for IA models. **(A)** Use of freely available large datasets (RNAseq, proteomics, metabolomics, CyToF) from patient materials to identify the pathways, genes, or processes of interest to the research question being investigated (e.g. reviewed in Buckley et al. 2021 [3]). **(B)** In vitro analysis of patient material in simple 2D culture systems (e.g., culturing fibroblasts on tissue culture plastic [99]), more complex 3D culture systems involving the incorporation of stromal cells or immune cells into gel structures (e.g., collagen or hydrogel) or the formation of 3D organoids [e.g. [100, 101],]. These types of culture systems are progressing towards more whole tissue models including the creation of mini-bones within tissue culture [102] or the use of organ-on-a-chip style microfluidics channels (e.g., reviewed in [98]). Figure created in Biorender.com.

Given that all IA models result in joint swelling, tenderness, and limited mobility, it is vital that researchers are aware of the direct impact housing conditions have on animal welfare. Several refinements in social housing, optimal environmental conditions (temperature, bedding, location of food/water) and handling of arthritic animals have been published previously and are summarised in **Figure 3** [55, 79, 81]. Substandard conditions increase the likelihood of animals exhibiting abnormal behaviours (e.g., aggression) and exhibiting signs of distress.

Imaging modalities can be used to visualise disease progression. This is increasingly the case in clinic, where ultrasound is becoming the standard of care for monitoring of synovial inflammation and patient response to treatment. *In vivo* microCT is a non-invasive x-ray tool that produces 3D, high resolution (up to ~5 micron, although ~15 micron is achievable in real-world situations with most current models) anatomical images, providing information about joint pathology in small-animal studies. Through this, longitudinal studies can be performed that assess the effect of treatment on factors, including bone quality and mass. Indeed microCT proved useful in tracking structural changes in tibial subchondral bone in a rat model of low dose monosodium iodoacetate induced osteoarthritis, as well as in tracking changes in bone during preclinical drug intervention [82]. Proulx et al [83]. showed that progression of joint erosion could be visualised over time in the TNF-Tg model of arthritis, and the authors were able to demonstrate that treatment with anti-TNF antibodies was able to prevent bone erosion of both talus and patella volumes.

An alternative imaging tool available to some arthritis research groups is MRI, which can readily discriminate between inflammation and bone destruction. Arthritis progression measured by MRI method has been shown, in the K/BxN serum transfer model of arthritis, to correlate well with clinical and histological progression [84]. To date, *in vivo* microCT and MRI are not widely or routinely used to study the bone impact of inflammatory arthritis, but the continuing improvements in speed and resolution, combined with increasing availability of scanners, are likely to result in an increase in such studies with time. Other *in vivo* imaging systems exist, such as the IVIS® Spectrum that combines 2D and 3D optical tomography. By using bioluminescent and fluorescent reporters across the blue to near-infrared wavelength region, disease progression, cell trafficking and gene expression patterns in living animals can be monitored. Using such tools, either as stand-alone or by combining approaches, allows for further refinements in IA studies and the use of longitudinal studies can reduce the total number of animals used (reduction), and be a refinement if they enable *in vivo* studies to be ended at an earlier timepoint during clinical progression. However, this must be balanced against the requirement for repeated anaesthesia of individual animals, which can lead to increased aversion and stress behaviours during the process [85].

### Ensuring appropriate reduction and robustness in experimental design

Randomised, double blinded control clinical trials (RCT) have highly defined study protocols, including the primary and secondary outcomes measurements, power calculations to achieve statistical significance, processes for blinding researchers and randomising patients into groups, all of which aim to ensure transparency and reproducibility of the data obtained. The “ARRIVE” guidelines, published in 2010 [86] and updated as “ARRIVE 2.0” in 2020 [87] set out the requirements for transparent and accurate reporting of *in vivo* studies. They aim to improve the standard of reporting and over time the standard of experimental design to address the reproducibility crisis in biomedical science [88–91]. The guidelines comprise a checklist of information for inclusion into any publication and are increasing becoming integrated into publisher’s author guidelines list to ensure transparency in study design and outcomes for all in the field. The basic minimum reporting requirements are akin to the minimum requirement for an RCT and are detailed in the “ARRIVE Essential 10” checklist, which includes providing sufficient details on the: 1. Study Design; 2. Sample size; 3. Inclusion and exclusion criteria; 4. Randomisation; 5. Blinding; 6. Outcome measures; 7. Statistical methods; 8. Experimental animals; 9. Experimental procedures and 10. Results.

The Experimental Design Assistant (EDA) tool [92] was developed by the National Centre for the 3Rs (a UK-based scientific organisation dedicated to developing and identifying 3Rs technologies and approaches) in response to findings that widespread errors in experimental and statistical design were apparent in published *in vivo* work [93]. It is freely available (https://eda.nc3rs.org.uk/) and aims to support researchers to comply with the ARRIVE guidelines by considering aspects, such as randomisation and blinding, at the experimental design stage and to improve reproducibility and statistical. Once experimental design using the EDA tool (or similar) is completed, the information contained within it can be used to aid consultation with local statisticians. Robust data from previous or pilot IA experiments, collected using the score sheets such as those described here (**Figure 1**) or similar, is invaluable when calculating experimental power, considering the multiple sources of variation endemic to these models (disease incidence, severity, and timing of arthritis onset, attrition of animals on extended study timelines, specific background, and genetic mutant strains during the study).

Use of the most state-of-the-art technologies can ensure that maximum information is generated from every experiment. One such technology is single cell RNA profiling, which offers a comprehensive transcriptome analysis at a single cell level. As each cell technically represents a biological replicate and thousands of cells can be processed per experiment, this advanced, phenotypic approach can generate large data sets and has the potential to describe complex tissue systems at a cellular and molecular level whilst reducing the number mice required for a robust analysis. Despite this, single cell RNA analysis is expensive and experimental design should be carefully considered prior to its use. To date, this technology has yet to be used to directly investigate transcriptional differences in the cellular composition of the synovium across difference phases of disease or in different IA models.

Analysis of single cell RNA from STIA (K/BxN serum transfer) synovial tissue has revealed complex heterogeneity within tissue resident fibroblasts [10], the presence of vascular-interacting and T cell-interacting fibroblast subtypes [13] and the alignment of such subtypes with those observed in human RA synovial biopsies [6, 10, 94]. Furthermore, these fibroblast gene signatures have been shown to positively correlate with treatment refractory RA (individuals that have failed multiple biological treatments) and may offer a new approach for therapeutic targeting [95, 96]. Similarly, single cell profiling has provided a detailed description of the resting synovial membrane in wildtype (control) C57BL/6J mice [11]. This study described a population of Trem2^+^ Cx_3_cr1^+^ tissue resident macrophages that form a tight barrier in the synovial lining layer and, under homeostatic conditions, provide immune privilege to the joint. Analogous tissue resident macrophages have also been observed in human joints and are thought to play an important role in re-establishing homeostasis and providing tolerance to RA flare [5].

### Replacement technologies: Moving towards an *in vitro* joint

**Figure 4.**
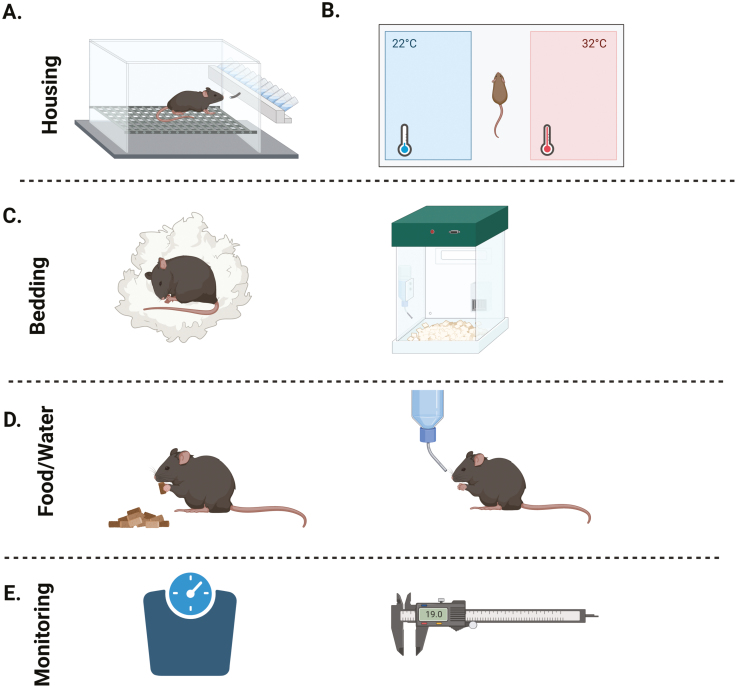
Refinement considerations regarding environment, housing, and choice of cohort. **(A).** Social housing with same sex and appropriate cage mates promotes social exploration and natural behavioural activities such as digging, but also provide social support during stressful situations. However, incompatible mice can lead to aggression, stress and injury which is more common in males. **(B).** Standard mouse housing conditions generally have room temperatures of between 20-24°C (68-75F) and are as stable as possible. Consideration could be given to increasing these temperatures for arthritic mice, as studies have shown that warmer temperatures are most preferred (described in detail in Hawkins et al. 2015 [55]). **(C).** Environmental enrichments provide sensory and motor stimulation. Soft, non-tangling nesting material, as well as soft litter reduce pain on walking, and cushion sore joints. **(D).** Easy access to food and water is necessary to cater for any disability in movement. This can be achieved by using bottles with long spouts and placing soft palatable food on the cage floor. **(E).** When handling animals avoid catching them by the tail, a practice known to induce a profound stress response. Instead, mice should be restrained with cupped hands or encouraged to enter handling tunnels [79, 80], this reduces stress and discomfort, which can be a potential source of variation within studies, while increasing willingness of the mice to interact with the observer. **(F).** Daily calliper measurement and weight measurements ensure that mice are carefully monitored and that disease course for each model is thoroughly understood by researchers and animal care staff. Figure created in Biorender.com.

Efforts to replace *in vivo* systems with complex *in vitro* constructs are continuing, and there are now systems available to model aspects of virtually every physiological process or organ system. These model systems range from 3-D self-organising mixed cell organoids through to microfluidic “organ-on-a-chip” methods or fabrication of tissue-like structures, using bioprinting and hydrogels. The expansion and progress of these techniques has been rapid over the past decade, but their application to studies of inflammatory arthritis remains limited (reviewed in detail [97, 98]) and summarised in **figure 4**. Inflammatory arthritides are not only multi-joint, nonetheless also multi-organ diseases that are dependent on the immune system and the circulatory system for their pathology. This has previously been used as an argument against the feasibility of modelling inflammatory arthritis *in vitro*. However, improved understanding of the multiple cell-cell interactions that occur within each of the tissues is now enabling *in vitro* modelling of certain aspects of these diseases. For example, control of leukocyte trafficking across the endothelium into the joint can be successfully modelled using microfluidics [103, 104], whilst 3-D organoids and on-chip models can be used to interrogate cell-cell communication pathways between stromal cell populations within the synovium [e.g. [12, 105, 106],]. A recent example of this approach used Matrigel organoids containing endothelial cells and fibroblasts to reveal that endothelial NOTCH3 ligands drives the spatial organisation of fibroblasts within the sublining layers [12]. The authors then used *in vivo* murine models to demonstrate that genetic deletion of *Notch3* reduced the clinical score and inflammatory infiltrate within the synovium, supporting previous findings that different populations of fibroblasts differentially drive damage in *in vivo* arthritis models [10]. Moreover, 3-D “synovium-on-a-chip” models have been developed to allow visualisation of TNFα-induced fibroblast organisation over 2 days and that support studies into cartilage-synovium cellular crosstalk [105, 106].

Advances in 3-D *in vitro* techniques are now allowing the maintenance of previously unculturable cell types, such as the osteocyte. These cells (the most numerous cell type within the bone) are notorious challenging to culture due to their requirement for a mineralised, collagenous 3-D environment. This specialised environment has now been recreated *in vitro* using a fibrin-containing hydrogel supported by brushite anchors, which provide strain and a source of calcium and phosphorous for mineralisation [102]. The ability to culture osteocytes bring the possibility of true ‘joint-on-a-chip’ models closer by allowing incorporation of all the relevant cell types.

Several of the methods described above allow the incorporation of precious, but extremely limited, patient material to realise the potential of humanised and/or personalised experimental model *in vitro* systems. Advances in imaging technologies combined with incorporation of patient material provides the opportunity to pre-screen treatment options and ultimately offers the possibility of precision medicine for patients based on the cellular and molecular processes underlying their disease pathology as well as reducing reliance on animal models of RA for discovery ­science.

## Concluding remarks

Huge strides are being made in the modelling of disease processes *in vitro,* presenting an opportunity to reduce reliance on *in vivo* models for many aspects of preclinical research. This replacement of *in vivo* methodologies with *in vitro*, should be the primary aim for any researcher wherever possible. However, *in vivo* modelling of inflammatory arthritis has been, and continues to be, crucial to understanding the aetiology and pathological progression of diseases, such as rheumatoid arthritis, and in developing and testing treatments for it. In parallel, clinical developments in arthritis assessment, monitoring, and pain management should inform *in vivo* experimental design and delivery. These developments will provide additional understanding that can support local ethical review bodies, licensing authorities and expert animal welfare officers and veterinarians when making decisions around humane endpoints. Thus, as *in vivo* research informs clinical practice, so developments in clinical practice, ethical frameworks, and advances in understanding of experimental design must inform research practices.

## Supplementary Material

kyac010_suppl_Supplementary_Figure_S1

kyac010_suppl_Supplementary_Figure_S2

## Data Availability

The data underlying this article will be shared on reasonable request to the corresponding author.

## Abbreviations

ACR: American College of Rheumatology
AIA: Antigen-induced arthritis
ARRIVE: Animal Research Reporting of In Vivo Experiments
Balb/c: Mouse strain
BSA: Bovine serum albumin
CIA: Collagen-induced arthritis
CD: Cluster of differentiation
C3H: Mouse strain
C57BL/6: Mouse strain
CT: Computed Tomography
CTLA: Cytotoxic T-lymphocyte-associated protein
CyToF: Cytometry Time of Flight
DMARD: Disease modifying anti-rheumatic drug
DBA: Mouse strain
EDA: Experimental Design Tool
EULAR: European League Against Rheumatism
G6PI: Glucose-6-phosphate isomerase
hTNF Tg: Transgenic mouse – expresses the Human TNF gene
IA: Inflammatory arthritis
ILR: Interleukin Receptor
JAK: Janus kinase
K/BxN: Transgenic mouse – cross between KRN (K) on C57BL/6 (B) background and NOD (N)
MHC: Major Histocompatibility Complex
MRI: Magnetic Resonance Imaging
NIH: United States National Institutes of Health
NOD: Non-obese diabetic mouse model
NOTCH: Neurogenic locus notch homolog protein
OVA: Ovalbumin
PROMS: National Patient Reported Outcome Measures
RA: Rheumatoid arthritis
RCT: Randomised Controlled Trial
STIA: Serum transfer-induced arthritis
TCR: T Cell Receptor
TNF: Tumour necrosis factor
TNF^ΔARE^: Transgenic mouse – deletion of ARE elements of the TNF gene
UKRI: United Kingdom Research and Innovation
VAS: Visual Analogue Scale
3-D: 3-dimensional
2-D: 2-dimensional
